# Uncommon presentation of a common arrhythmia

**DOI:** 10.1002/ccr3.3763

**Published:** 2021-01-12

**Authors:** Thierry Verbeet, Alexandre Almorad, Thomas Nguyen, Maurice Jottrand, José Castro

**Affiliations:** ^1^ University Hospital Brugmann Free University of Brussels Brussels Belgium

**Keywords:** absent retrograde conduction, atrioventricular reentrant nodal tachycardia, simultaneous dual ventricular responses

## Abstract

This case report demonstrates that atrioventricular and ventricular atrial conduction at rest may be unreliable in assessing the presence of reentrant atrioventricular nodal tachycardia.

## Graphical abstract

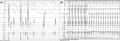

## 1 CASE REPORT

A 46‐year‐old lady complained of recurrent paroxysmal tachycardia during physical exercise.

**FIGURE 1:**
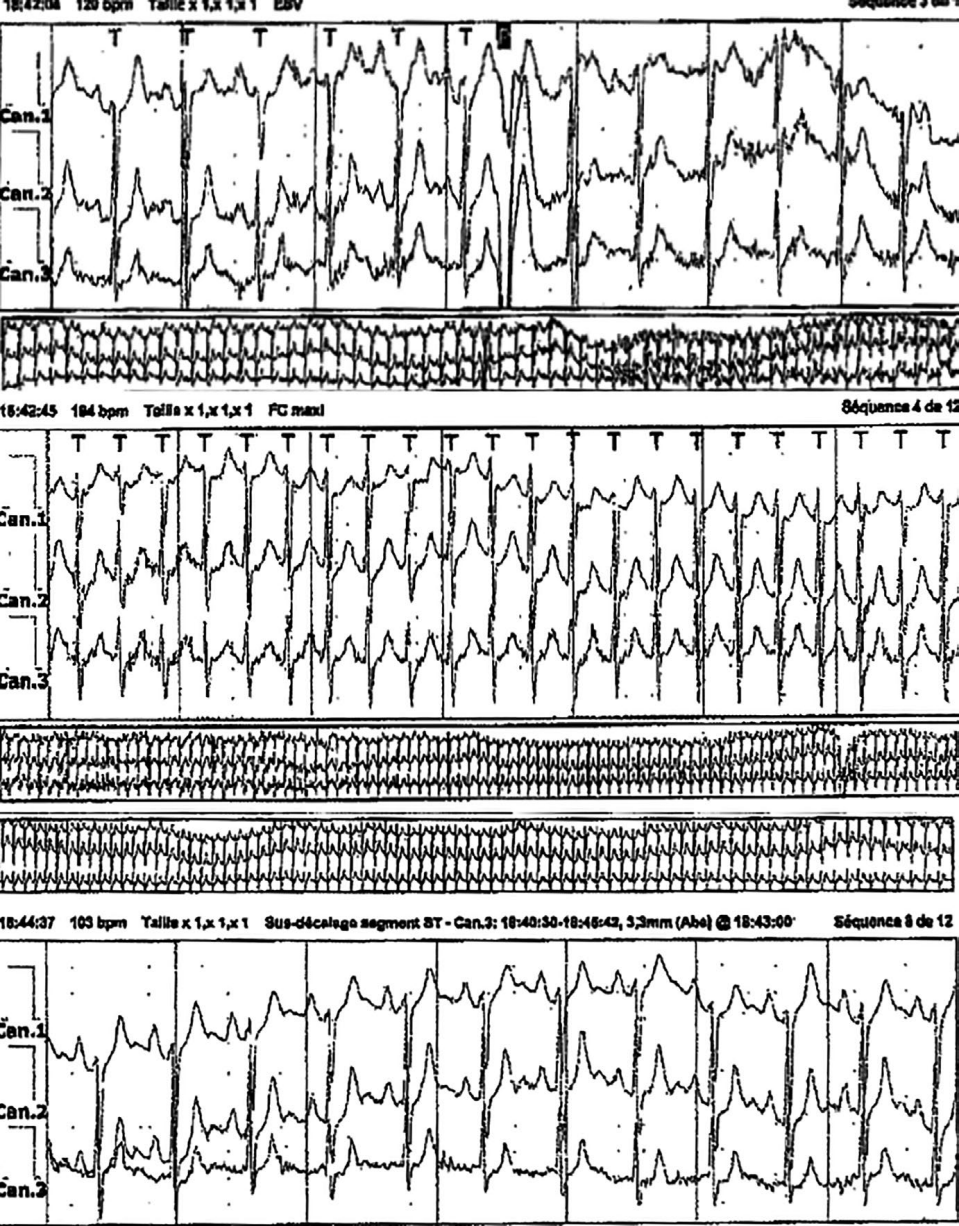
Upper tracing: sinus rhythm at exercise (120 bpm) (plus one VPB), before the occurrence of the clinical tachycardia. Middle tracing: supraventricular tachycardia. No P waves are seen. Lower tracing: spontaneous restoration of sinus rhythm after the exercise is stopped. P waves can be clearly seen. The ECG during tachycardia is compatible with the common form of atrioventricular nodal tachycardia. 25 mm/s

The tachycardia could be seen on an event recorder (Figure 1). It was a narrow QRS tachycardia reaching a rate of 194 bpm.

On the base of the tracing, a presumptive diagnosis of typical slow‐fast atrioventricular nodal tachycardia was done.

Medical treatment consisted of sertraline and omeprazole.

**FIGURE 2:**
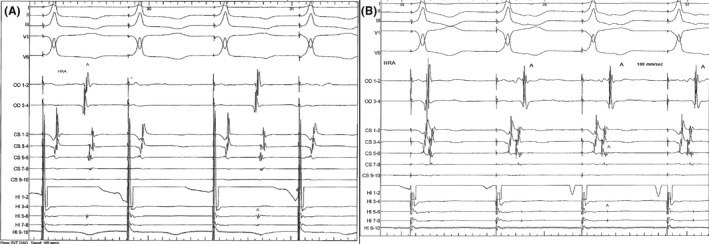
A, Para‐hissian ventricular pacing at baseline. There is retrograde block. B, During isoprenaline infusion, a sustained concentric retrograde conduction appears. Electrodes CS 5‐6 are situated at the level of the coronary sinus os

An electrophysiological study was performed. Para‐hissian ventricular pacing at baseline revealed absolutely no retrograde conduction (Figure 2A). Atrial pacing revealed an anterograde Wenckebach phenomenon at 700 ms atrial pacing cycle length and no anterograde nodal jump. After isoprenaline infusion (1 µg/min), retrograde dissociation persisted but at 2 µg/min one‐to‐one concentric retrograde conduction could be seen (Figure 2B). At 3 µg/min isoprenaline infusion, ventricular premature stimulation induced isolated narrow QRS ventricular echoes following progressive prolongation of the retrograde conduction by 65 ms with unchanged pattern of retrograde conduction. This suggested concealed retrograde block in the slow pathway with a slow‐fast ventricular echo.

**FIGURE 3:**
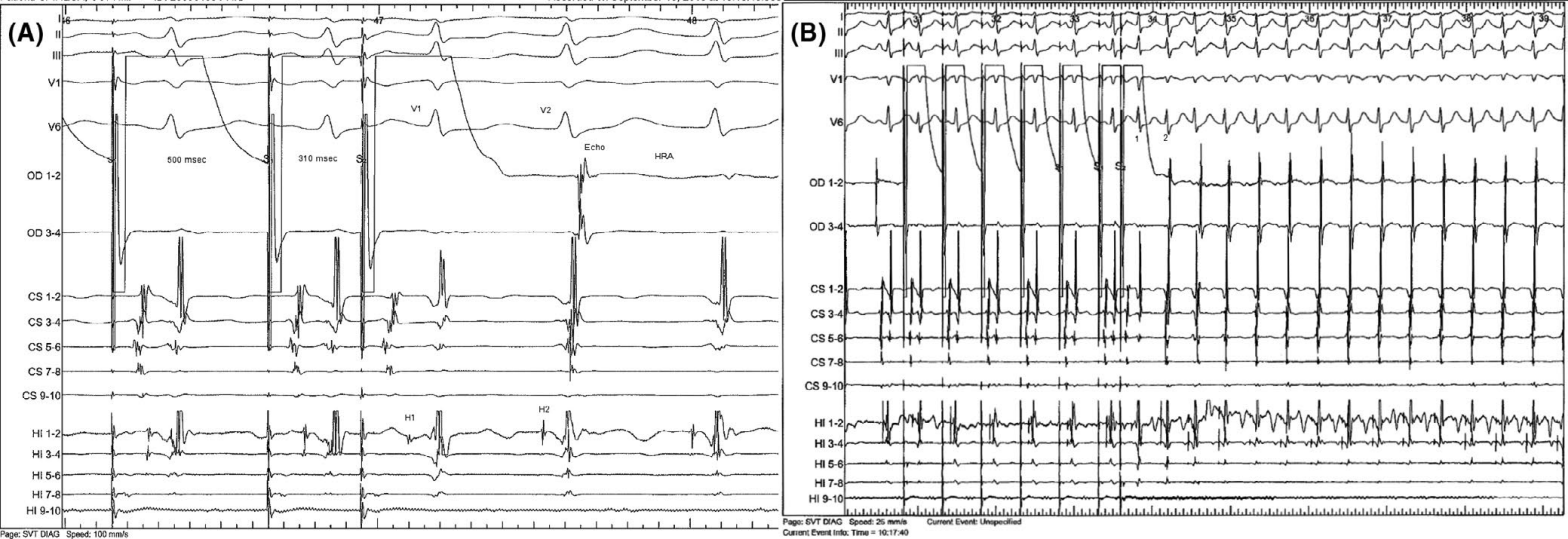
A, One typical atrial echo (Echo) is induced by a premature atrial stimulus (coupling of 310 ms) following a double ventricular response: through the fast nodal pathway (V1, AH interval: 155 ms) and through the slow nodal pathway (V2, AH interval: 590 ms). A second ventricular echo is visible but blocks retrogradely in the fast pathway. B, A typical sustained slow‐fast atrioventricular nodal tachycardia is induced after a double ventricular response (1 and 2) at a rate of 155 bpm

Atrial pacing induced a double ventricular response, one through the fast nodal anterograde pathway and one through the anterograde slow pathway followed by one retrograde fast pathway echo and a second ventricular echo with retrograde block in the fast pathway (Figure 3A). A typical episode of slow‐fast atrioventricular nodal reentrant tachycardia at 152 bpm could then be induced via the double‐response mechanism (Figure 3B).

The atrial activation during tachycardia was identical as the one during ventricular pacing when retrograde conduction was present, and it was not possible to advance the atrial electrogram when a ventricular pacing impulse was delivered during the refractory period of the His confirming the mechanism of the tachycardia. Five cryoablation lesions were delivered on the triangle of Koch on sites with an A/V ratio <1 and where rapid potentials suggesting the presence of the slow pathway were present. These lesions were delivered at the level of the upper part of the coronary sinus os and up to midway between the His recording and the coronary sinus os.

This treatment abolished the conduction through the slow pathway with disappearance of the double response and also of the isolated nodal echoes induced during ventricular pacing on isoprenaline.

After isoprenaline was stopped, all retrograde conduction disappeared again.

There was no arrhythmia recurrence after a follow‐up of 2 years.

## 2 DISCUSSION

Atrioventricular nodal reentrant tachycardia is typically induced with anterograde block over the fast pathway and conduction over the slow pathway with subsequent retrograde conduction over the fast pathway.^1^

Complete absence of retrograde conduction at baseline is infrequent in the setting of reentrant atrioventricular nodal tachycardia. Complete absence of retrograde conduction at the baseline usually favors the diagnosis of atrial tachycardia.

In previously described cases, the retrograde conduction can be absent during tachycardia^2^, ^3^, ^4^ or absent at baseline without inducible tachycardia and present under isoprenaline with inducible tachycardia.^5^, ^6^

Dual ventricular response during premature atrial stimulation inducing reentrant atrioventricular nodal tachycardia is another infrequent finding.^7^, ^8^, ^9^

It means that the fast anterograde pathway does not need to block anterogradely in order to trigger tachycardia. Very slow anterograde conduction through the slow nodal pathway finds the retrograde limb of the fast pathway excitable triggering slow‐fast reentry. During anterograde fast pathway conduction, a retrograde block is likely to be present on the slow pathway preventing retrograde concealed conduction at that level. The two observations, dual anterograde response and absence of retrograde conduction at baseline, are probably linked: bad retrograde conduction through the slow pathway is likely to be a prerequisite for dual response.

Hence, complete absence of retrograde conduction and/or no anterograde jump at rest does not rule out the diagnosis of atrioventricular nodal reentrant tachycardia. In fact, atrioventricular and ventriculoatrial conduction at rest is unreliable in assessing the presence of reentrant atrioventricular nodal tachycardia.

Isoprenaline infusion at increasing dosage should be used in these cases especially if tachycardia only occurs during exercise as was the case in this patient. Occurrence of the tachycardia during exercise also explains why the spontaneous tachycardia rate was also 42 bpm higher than the induced tachycardia.

The dual response demonstrates that absence of anterograde fast pathway block during premature atrial stimulation does not rule out atrioventricular nodal tachycardia.

Isoprenaline infusion allowed the tachycardia to be induced. Care should be taken when a high dosage is used because many side effects may occur.^10^

The situation described in this case report is different than the so‐called nonreentrant form of tachycardia due to dual ventricular response in sinus rhythm^11^ in which there is simultaneous anterograde conduction through the fast and through the slow pathway leading to one sinus beat giving two ventricular activations and hence tachycardia, although both arrhythmias are treated by slow pathway ablation. It is uncertain whether our patient could also suffer of nonreentrant tachycardia given the fact that at baseline very slow conduction in the slow anterograde pathway is a prerequisite. In our case, this slowing occurred only during the delivery of premature atrial stimulation.

## 3 CONCLUSION

This patient with typical atrioventricular slow‐fast reentrant nodal tachycardia showed a combination of electrophysiological features making it a very uncommon case: absence of baseline retrograde conduction and induction of tachycardia after a dual anterograde nodal response.

This case report demonstrates that atrioventricular and ventricular atrial conduction at rest may be unreliable in assessing the presence of reentrant atrioventricular nodal tachycardia.

## CONFLICT OF INTEREST

None declared.

## AUTHOR CONTRIBUTION

TV: served as main electrophysiologist. JC: served as main electrophysiologist. TN, AA and MJ: served as attending electrophysiologists.

## Data Availability

Data are available in article supplementary material.
